# Modified RCTU Score: A Semi-Quantitative, Visual Tool for Predicting Alzheimer’s Conversion from aMCI

**DOI:** 10.3390/brainsci14020132

**Published:** 2024-01-27

**Authors:** Ari Chong, Jung-Min Ha, Ji Yeon Chung, Hoowon Kim, IL Han Choo

**Affiliations:** 1 Department of Nuclear Medicine, School of Medicine, Chosun University/Chosun University Hospital, Gwangju 61452, Republic of Korea; arichong.md@gmail.com; 2 Department of Neurology, School of Medicine, Chosun University/Chosun University Hospital, Gwangju 61452, Republic of Korea; jiyeoni@chosun.ac.kr (J.Y.C.); hoowon@chosun.ac.kr (H.K.); 3 Department of Neuropsychiatry, School of Medicine, Chosun University/Chosun University Hospital, Gwangju 61452, Republic of Korea; ilhan.choo@chosun.ac.kr

**Keywords:** Alzheimer’s disease, amyloid burden, mild cognitive impairment, positron emission tomography, RCTU

## Abstract

This research evaluated the modified RCTU score, derived from amyloid PET scans, for predicting the progression from amnestic Mild Cognitive Impairment (aMCI) to Alzheimer’s Disease (AD). aMCI patients underwent baseline evaluations, including amyloid PET. AD conversion was identified through neuropsychological tests after observation. The RCTU was modified by segmenting frontal, parietal, and temporal lobes into left and right, resulting in seven areas. Scores from both modified and conventional RCTU were analyzed and compared. Among 45 patients, 12 progressed to AD (over 17.8 ± 6.8 months). AD converters showed higher scores in modified RCTU scores. Modified RCTU score had strong correlations with amyloid SUVR (*r* > 0.7). Modified RCTU sum score was the significant covariate of AD conversion. Modified RCTU could determine the asymmetry of amyloid deposits. We demonstrated that symmetric deposits of amyloid showed a higher risk for AD conversion when analyzed using modified RCTU. The modified RCTU score is a promising method for predicting AD conversion, correlating strongly with amyloid SUVR.

## 1. Introduction

Alzheimer’s disease (AD) is a common cause of dementia, and it affects 24% to 33% of adults aged older than 85 years [1]. The excessive accumulation of misfolded β-amyloid and neurofibrillary tangles are neuropathological hallmarks of AD [2].

Mild cognitive impairment (MCI) is an intermediary condition between normal cognitive status and dementia [3,4]. However, not all patients with MCI progress to AD. The conversion rate from amnestic MCI (aMCI) to AD is reported to be approximately 10% to 15% per year [5]. Patients with high amyloid retention have an 82% conversion rate to AD during a 3-year follow-up [6]. Medical intervention during the aMCI stage may prevent or slow the conversion to a full-blown AD [4].

Neuroimaging with positron emission tomography (PET) is used for early diagnosis and differentiation of various types of dementia [7,8,9]. Glucose metabolism can be assessed using ^18^F-fluorodeoxyglucose (F-18 FDG) PET, while amyloid deposition can be assessed using amyloid tracers such as ^18^F-florbetaben (F-18 FBB), ^11^C-Pittsburgh Compound B (PiB), ^18^F-flutemetamol, or ^18^F-florbetapir. Many studies have analyzed patient conversion from MCI to AD [2,10,11,12,13,14,15,16].

Quantitative variables like standardized uptake value ratios (SUVRs) are used to analyze PET images, but they are not convenient in routine clinical practice. Visual analysis of amyloid on PET using an FBB tracer employs a brain amyloid plaque load (BAPL) scoring system based on a conventional regional cortical tracer uptake (RCTU) score [17,18]. However, this scoring system has three predefined categories (i.e., negative/moderate/severe) and does not provide information on the sides or locations of amyloid deposition.

Therefore, we modified the conventional RCTU scoring system and investigated whether modified RCTU variables could predict the conversion of aMCI to AD in patients.

## 2. Materials and Methods

### 2.1. Patients and Study Overview

**Figure 1:**
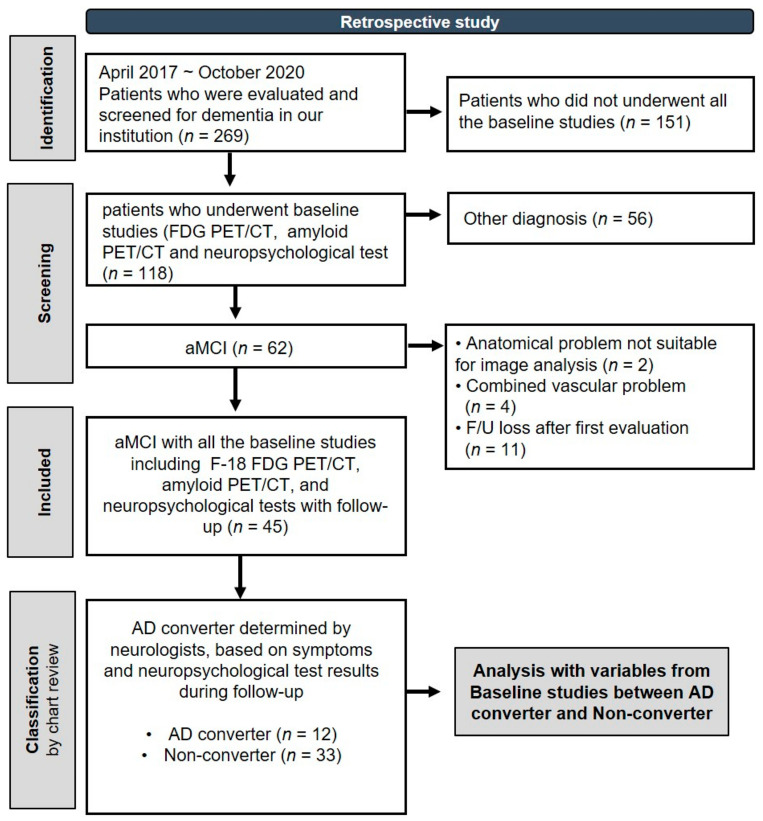
Flow Diagram Depicting the Study Design.

The present study was conducted retrospectively, spanning from April 2017 to October 2020. During this period, a total of 269 patients from the community and local dementia clinics were evaluated and screened for dementia at our center. Among these patients, we only enrolled those who had amnestic mild cognitive impairment (aMCI) and who underwent all the baseline studies, including F-18 FDG PET/computerized tomography [CT], amyloid PET/CT, and neuropsychological tests. Figure 1 is a flow diagram depicting the study design. The diagnosis of aMCI was established by a neurologist during the patient’s initial visit, based on clinical symptoms and the results from the Seoul Neuropsychological Screening Battery (SNSB) [19]. Petersen’s criteria [20] was used for the diagnosis of aMCI, with a requisite z-score of less than −1.5 in the memory domain, adjusted for age, education, and gender norms. At the time of aMCI diagnosis, the clinician was not informed of the amyloid PET scan results. We excluded individuals who did not follow up or had cerebrovascular disease or space-occupying lesions in the brain. In total, 45 patients with aMCI were included in the final analysis. After follow-up, our team of neurologists (HWK and JYJ) determined AD conversion and non-conversion based on the patient’s symptoms and neuropsychological test results. For the purpose of identifying AD converters, the diagnosis was determined based on the National Institute on Aging and the Alzheimer’s Association (NIA-AA) criteria [21,22]. At the time of evaluating AD conversion, the assessors were aware of the amyloid PET information.

We conducted conventional and modified RCTU scoring based on amyloid PET/CT and analyzed the inter-rater agreement of RCTU scoring. We then compared the RCTU scores between the AD conversion and non-conversion groups and analyzed the correlation, odds ratio, and cut-off value of RCTU variables for predicting AD conversion from aMCI. Additionally, we analyzed the correlation between conventional and modified RCTU variables, FDG variables, and neuropsychological variables.

### 2.2. F-18 FBB PET/CT Scanning

A brain PET/CT scan was conducted using a 3D time-of-flight-based PET/CT scanner (Discovery MIDR, GE Healthcare, Milwaukee, WI, USA) 90 min after intravenous injection of F-18 FBB (300 MBq). The imaging acquisition lasted for 20 min, during which the scanner was set to the following specifications: 120 KeV, 155 mA, a thickness of 3.75 mm, a field of view (FOV) of 250 mm, and a matrix size of 256 × 256.

### 2.3. Variables of Amyloid PET/CT Data

In this study, we utilized three different amyloid variables: (1) a simple visual inspection that categorized patients as either positive or negative, (2) conventional RCTU and BAPL scoring, and (3) modified RCTU scores and their corresponding sum total. To ensure accuracy, all decisions regarding amyloid variables were made by two nuclear medicine physicians (JMH and AC) with 10 and 11 years of experience in their respective medical specialties. Inter-rater agreement was subsequently analyzed. The raters of the amyloid PET were blinded to the patient classification.

The conventional RCTU scoring system is based on the assessment of tracer uptake in three grades for each of the regions mentioned earlier (1 = no tracer uptake, 2 = moderate tracer uptake, and 3 = pronounced tracer uptake). The cortical regions evaluated in the conventional RCTU scoring system include the lateral temporal cortex, frontal cortex, parietal cortex, and posterior cingulate cortex/precuneus (PC2). The BAPL scoring system, on the other hand, is based on the RCTU scores and utilizes three grades (1 = no amyloid load, all RCTU is scored 1; 2 = minor amyloid load, if there is any RCTU, the lesion is scored 2; and 3 = significant amyloid load, if there is any RCTU, the lesion is scored 3 points) [17,18]. The scoring in BAPL is conducted through only visual inspection. The distinction between scores 2 and 3 is determined based on whether the amyloid load involves a small region or the entire region, respectively. The conventional RCTU sum is calculated by summing up the total RCTU scores of each of the four regions. Given that the conventional RCTU score can range from 1 to 3 points across four regions, the total possible score spans from a minimum of 4 to a maximum of 12 points.

We modified the conventional RCTU system by dividing each area into left and right regions except for PC2. Consequently, we used a total of seven regions: the left lateral temporal cortex, right lateral temporal cortex, left parietal cortex, right parietal cortex, left frontal cortex, right frontal cortex, and PC2. Additionally, we used the sum of RCTU scores from all seven regions instead of BAPL. If the amyloid scan was negative, each of the RCTU scores of the seven regions was scored as 1 point. The modified RCTU sum scores can range from 7 to 21 points. The sum of modified RCTU scores (modified RCTU sum) for a positive scan could range from 8 to 21 points. A negative amyloid scan had the lowest modified RCTU sum of seven points. Scans with a total score greater than or equal to eight points were considered positive.

To evaluate the asymmetry of amyloid accumulation in both the left and right cerebral hemispheres, we utilized the ‘right sum of RCTU’ scores, which is the combined total of the scores from the right frontal, right parietal, and right temporal regions. Similarly, the ‘left sum of RCTU’ was calculated using the corresponding regions in the left hemisphere. The asymmetry of amyloid deposition was determined based on the difference in the sum of modified RCTU scores of the right and left sides. Among patients with a positive amyloid PET, those with a non-zero value for the difference between the left and right modified RCTU sum scores were defined as having ‘asymmetric amyloid deposition’. Conversely, a value of zero indicated ‘symmetric amyloid deposition’.

### 2.4. Measurement of Amyloid SUVR

The SUVR of amyloid was measured by MIM Neuro Software (version 6.9.7; MIM Software Inc., Cleveland, OH, USA), with the cerebellum used as the reference region. In addition to the whole brain, the SUVR was also quantified in bilateral frontal, parietal, and temporal lobes. The lobe configurations were customized for our institution by MIM Software Inc.

### 2.5. Other Clinical Variables

The study incorporated several clinical variables such as patient age, sex, left- or right-handedness, education (in years), and the status of apolipoprotein E4 (apoE4). The apoE4 status was assessed in two different manners: (1) by distinguishing between apoE4 carriers and non-carriers and (2) by classifying individuals as apoE4 homozygotes, heterozygotes, or non-carriers.

### 2.6. Statistical Analysis

The inter-rater agreement for RCTU scoring between the two readers was analyzed. A statistical analysis of the AD conversion and non-conversion groups was conducted using either a Student *t*-test or Mann-Whitney U test (M-W test), based on the results of a normality test (Shapiro-Wilk test) to identify any statistically significant differences in the variables between groups. We performed a partial correlation test between AD conversion and each modified or conventional RCTU variable. Fisher’s exact test was used to compare the proportion of AD converters between patients with asymmetric and symmetric amyloid deposits in amyloid PET-positive individuals. We plotted a Kaplan-Meier graph to observe AD converter incidence between baseline amyloid PET-positive and negative groups. Additionally, for amyloid PET-positive patients, a second graph was created to classify amyloid deposition as symmetric or asymmetric based on modified RCTU evaluations to study its effect on AD conversion. Cox proportional hazard regression was conducted. Receiver operating characteristic curve (ROC) analysis was conducted, and a comparison of ROC was conducted. Logistic regression analyses were performed, revealing differences between groups. A *p*-value of less than 0.05 was considered statistically significant. IBM SPSS statistics for Windows, version 26.0 (IBM Corp., Armonk, NY, USA; released 2019) and MedCalc^®^ Statistical Software, version 20.007 (MedCalc Software Ltd., Ostend, Belgium; released 2021) were used for data analysis and graphs.

## 3. Results

### 3.1. Patient’s Characteristics

A total of 45 patients with aMCI (76.24 ± 7.12 years, 26 males (57.8%)) were included in the study. Patient characteristics are listed in Table 1, with 17 patients having positive and 28 patients having negative amyloid PET/CT scan findings.

### 3.2. AD Converter Group and Non-Converter Group

After an average follow-up period of 23.62 ± 9.11 months (ranging from 11.6 to 41.3 months), patients were divided into two distinct categories: the AD converter group (*n* = 12) and the non-converter group (*n* = 33). The specific characteristics of each group are outlined in Table 2. The time to AD conversion diagnosis from the first visit was 17.8 months (range from 12.1 to 30.1 months). Based on the simple visual interpretation of the amyloid PET scans, seven patients in the AD converter group exhibited positive results, while five showed negative. In contrast, the non-converter group included 10 patients with positive results and 23 with negative. However, the simple visual analysis of the amyloid PET scans did not reveal a statistically significant difference between the two groups (*p* = 0.086). Between groups, conventional and modified RCTU variables were different (Table 2).

The other laboratory and clinical variables, such as patient age, sex, follow-up interval, education, and apoE4 status, did not show significant differences between the AD converter and non-converter groups. The time to AD conversion diagnosis showed no significant correlation with age, education, amyloid SUVR, conventional RCTU, modified RCTU, or clinical variables.

### 3.3. Conventional and Modified RCTU Sum Scores

**Figure 2:**
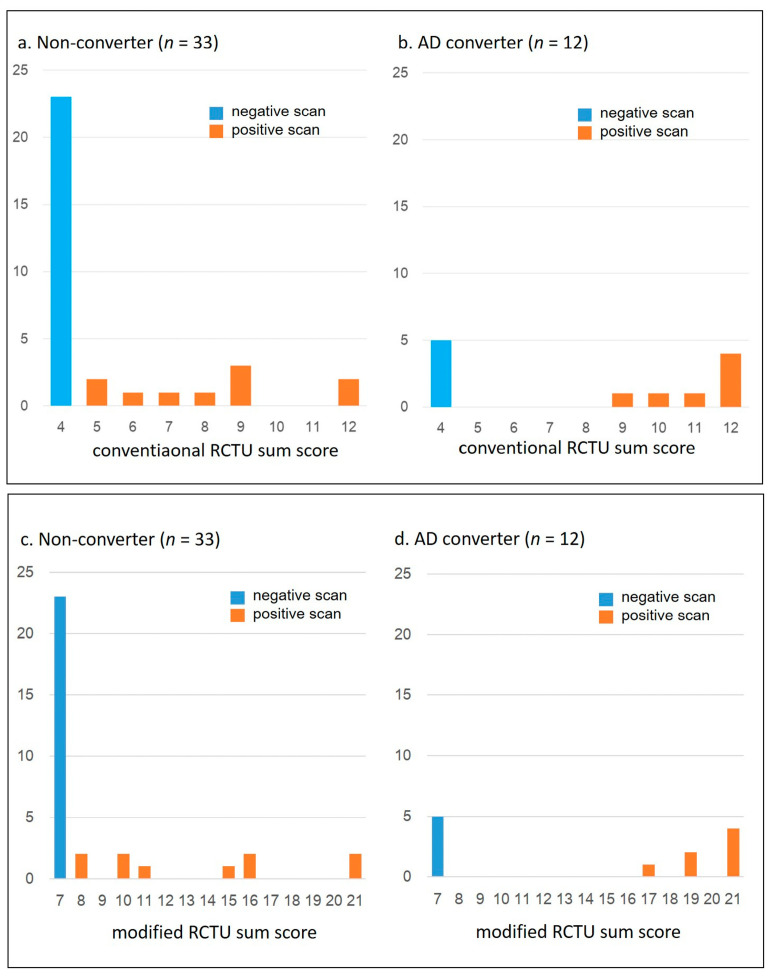
Distribution of Conventional and Modified RCTU Sum Scores in AD Converters and Non-converters. This figure depicts the distribution of conventional and modified RCTU sum scores in two distinct groups: Alzheimer’s disease (AD) converters and non-converters. The *y*-axis represents the frequency of subjects falling within each score range. (**a**,**b**) demonstrate the distribution of the sum of conventional RCTU scores in each group. Given that the conventional RCTU score can range from 1 to 3 points across four regions, the total possible score spans from a minimum of 4 to a maximum of 12 points. Mann-Whitney U (M-W) test indicated a significant difference in the conventional RCTU sum scores between AD converters and non-converters (*p* = 0.0197). (**c**,**d**) depict the distribution of the sum of modified RCTU scores in each group. The modified RCTU sum scores can range from 7 to 21 points. The M-W test showed a significant difference in the modified RCTU sum scores between the two groups (*p* = 0.018).

Figure 2 illustrates the distribution of RCTU sum scores in AD Converters and non-converters. A significant difference was observed in the modified RCTU values between the two groups (*p* = 0.018). Both the sum scores of conventional RCTU scores and modified RCTU scores were statistically different between groups by the M-W test (*p* = 0.02 and 0.018, respectively).

### 3.4. Correlation between RCTU Scores and Amyloid Deposits

**Figure 3:**
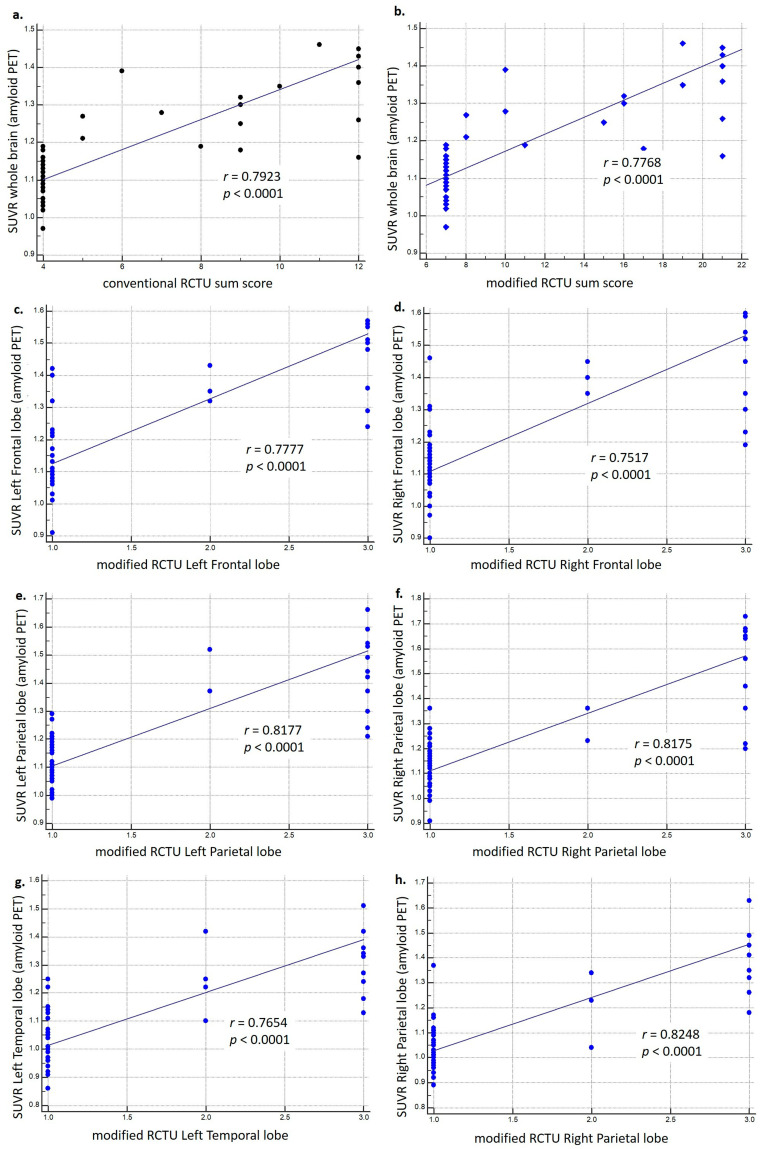
Correlation of Amyloid Deposits (SUVR) with Conventional and Modified RCTU Scores. These figures show cases scatter diagrams depicting the correlation of amyloid deposits (quantified by MIM software) with both conventional and modified RCTU scores. The trend lines are shown as reduced major axis lines. (**a**) The analysis with conventional RCTU sum scores exhibits a correlation coefficient (r) of 0.7923, indicating a significant correlation (*p* < 0.0001, 95% CI: 0.65 to 0.8810) with whole brain SUVR. (**b**) The analysis with modified RCTU sum scores displays a correlation coefficient (r) of 0.7768, likewise demonstrating a significant correlation (*p* < 0.0001, 95% CI: 0.6260 to 0.8716) with whole brain SUVR. (**c**–**h**) display graphs that illustrate the correlation between the modified RCTU values of the left and right lobes and the amyloid SUVR values of the corresponding lobes.

We examine the associations between both the conventional and modified RCTU sum scores and the amyloid deposits, as measured by whole-brain SUVR. As depicted in Figure 3, both sum scores show significant correlations with the amyloid deposits, with each achieving a correlation coefficient (r) greater than 0.7. The statistical significance of these correlations is confirmed by a *p*-value of less than 0.0001 in both analyses.

Further, we delved into the specific associations between the amyloid SUVRs of distinct brain lobes and their corresponding modified RCTU scores. Upon conducting an analysis using Spearman’s rho, we identified robust positive correlations across all considered lobes of the brain. To elaborate, we observed correlation coefficients (r) for the left frontal, left parietal, left temporal, right frontal, right parietal, and right temporal lobes, which were recorded as 0.733, 0.772, 0.723, 0.718, 0.744, and 0.736, respectively (all *p* < 0.0001). These findings underscore the significant positive relationships between the amyloid SUVRs of individual lobes and their corresponding modified RCTU scores.

In summary, these findings indicate robust correlations between the amyloid SUVR in each brain lobe and their respective modified RCTU scores, suggesting a significant association between RCTU scores and amyloid deposits.

### 3.5. Asymmetry of Amyloid Deposition

The asymmetry of amyloid deposition was determined by the difference in the modified RCTU values of the left and right lobes, as described in Section 2.3. Out of the total patients, 17 were amyloid PET-positive. Among these, nine showed asymmetry of amyloid deposition, while 8 had symmetric amyloid deposits. Of the nine with asymmetric deposits, one became an AD converter, whereas among the eight with symmetric deposits, six converted to AD. Patients with symmetric deposits converted to AD significantly more often (*p* = 0.0152). There were cases where one lobe was amyloid negative while the other was positive: two cases with the right lobe negative and left lobe positive, and two cases with the right lobe positive and left lobe negative. None of these four cases converted to AD.

Logistic regression showed that as it progresses from no amyloid deposit, to unilateral deposit, and then to bilateral deposit, the risk of AD conversion increases (odds ratio 3.1481, 95% CI 1.3070 to 7.5830).

### 3.6. Kaplan-Meier Survival Analysis

**Figure 4:**
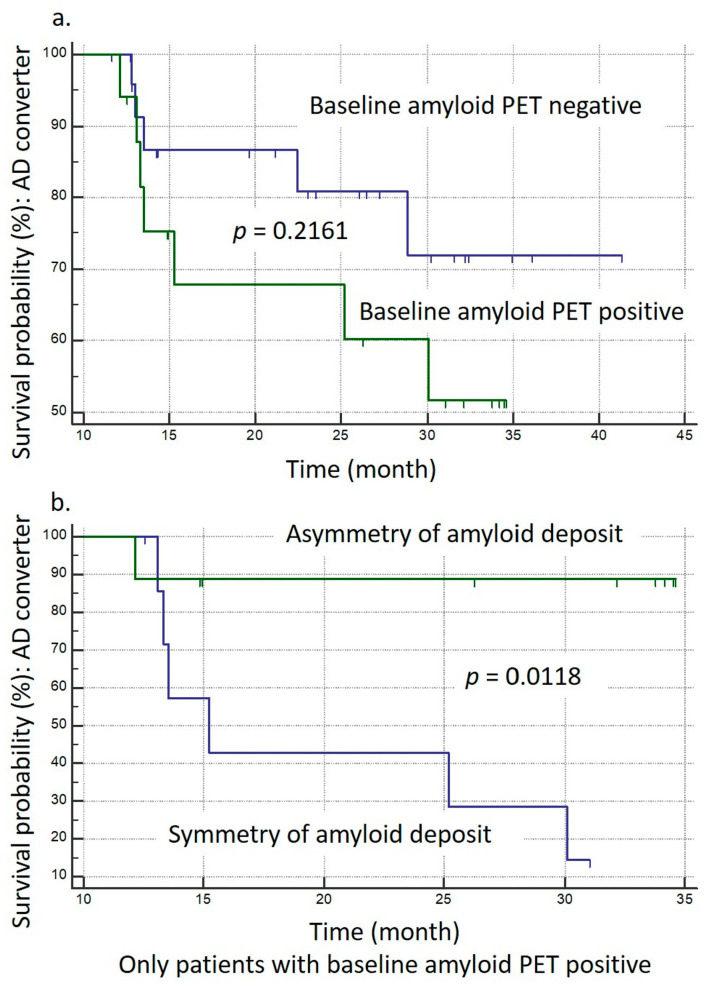
Kaplan-Meier Survival (K-M) Analysis. Panel (**a**): K-M analysis comparing AD conversion among amyloid PET-positive and negative patients. Panel (**b**): K-M analysis for amyloid PET-positive patients, differentiating groups based on the symmetry and asymmetry of amyloid deposits, as determined by the left-right difference in modified RCTU scores.

We conducted two Kaplan-Meier survival analyses (K-M analysis), defining ‘event’ as the occurrence of AD conversion. Figure 4a shows the results. The first analysis was conducted with amyloid PET-positive and negative patients. It appears that amyloid PET-positive patients have different conversion timings compared to the PET-negative group. This is evident from the mean survival times: 26.714 months for PET-positive and 35.344 months for PET-negative groups. However, the Logrank test for comparing survival curves shows a *p*-value of 0.2162, which suggests that the difference between the two groups is not statistically significant at conventional levels (*p* = 0.2162).

Figure 4b shows the second analysis with amyloid PET-positive patients only. Among them, asymmetry and symmetry deposits of amyloid showed results that indicate a substantial distinction between those with symmetric and asymmetric amyloid deposits. The symmetric group had six events (75%) and two censored cases (25%), whereas the asymmetric group had one event (11.11%) and eight censored cases (88.89%). The average survival time for the symmetric group was 20.21 months (95% CI: 14.546 to 25.873), with the median at 15.233 months (95% CI: 13.067 to 30.100), while the median survival time for the asymmetric group was not provided. The Logrank test yielded a chi-squared value of 6.3407 with a significant *p*-value of 0.0118, suggesting a statistically significant difference in survival times between the two groups. Hazard ratios (HR) indicated a lower risk in the symmetric group (HR: 0.1405, 95% CI: 0.03049 to 0.6473) and a higher risk in the asymmetric group (HR: 7.1189, 95% CI: 1.5450 to 32.8023). These findings demonstrate a crucial difference in AD conversion between patients with symmetric and asymmetric amyloid deposits, with Section 3.5 indicating that the symmetric group, which had more events, exhibited a higher risk of AD conversion compared to the asymmetric group.

### 3.7. Cox Proportional Hazards Regression

In the Cox proportional hazards regression analysis, we included time to AD conversion diagnosis (accounting for the observation period for censored case), final AD conversion status, and covariates such as age, education, sex, amyloid SUVR, and RCTU sums (both conventional and modified). Utilizing a forward conditional method, the analysis revealed that only the modified RCTU sum significantly predicted outcomes, leading to the exclusion of other variables. The modified RCTU sum’s hazard ratio was 1.1318 (95% CI: 1.0309 to 1.2426, *p* = 0.0093), demonstrating its predictive strength. The conventional RCTU sum lacked significance and was removed. The model’s overall chi-squared value stood at 6.516 (*p* = 0.0107), and its predictive accuracy, gauged by the AUC or C-index, was 0.703 (95% CI: 0.549 to 0.830), underscoring the modified RCTU sum’s relevance in AD conversion risk assessment.

### 3.8. ROC Analysis

**Figure 5:**
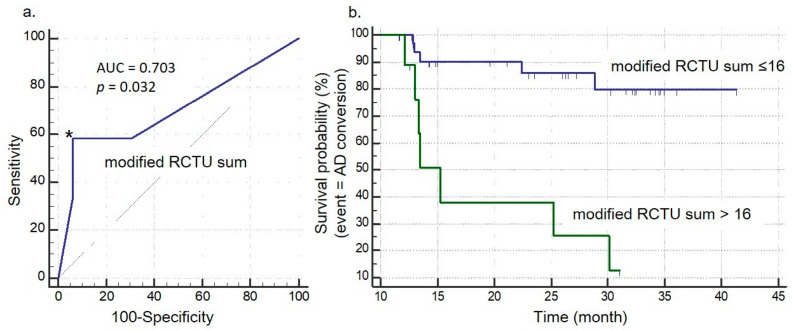
ROC Analysis and Survival Probability using Conventional and Modified RCTU Sum Scores. Panel (**a**) displays the Receiver Operating Characteristic (ROC) analysis of the modified RCTU sum scores. With a cut-off value of 16 (* in the graph), the ROC curve (blue line) indicates a sensitivity of 58.3%, specificity of 93.9%, and an Area Under Curve (AUC) of 0.703 (*p* = 0.032) for predicting Alzheimer’s Disease (AD) conversion from Amnestic Mild Cognitive Impairment (aMCI). Panel (**b**) shows the survival probability based on K-M analysis, divided by the modified RCTU sum score cut-off of 16. The graph represents the comparison of survival probabilities (or the risk of AD conversion) between patients with RCTU sum scores equal to or less than 16 (blue line) and those with scores greater than 16 (green line).

Figure 5 illustrates the survival probability of AD conversion and the result of ROC analysis. On ROC analysis, a modified RCTU sum over 16 showed a sensitivity of 58.3%, specificity of 93.9%, and an area under the curve (AUC) of 0.703 (*p* = 0.032) for AD conversion from aMCI.

ROC analysis was conducted using the conventional RCTU sum, yielding an AUC of 0.699 (*p* = 0.034) for predicting AD conversion. A comparison of ROC curves between the modified RCTU sum and the conventional RCTU sum was performed. Although the AUC for the modified RCTU sum was slightly higher at 0.703 versus 0.699, this difference was not statistically significant (*p* = 0.3827).

### 3.9. Agreement

Two readers showed very good agreement in reading and scoring the conventional and modified RCTU variables (weighted kappa ranged from 0.9 to 1).

## 4. Discussion

Our research provides valuable insights into the utility of the modified RCTU score as a predictor of AD conversion from aMCI. (1) We discovered a strong correlation between the modified RCTU scores and the standard quantitative measure, amyloid SUVR (Figure 3). These findings suggest that the modified RCTU sum score may serve as a semi-quantitative, visual scoring tool for amyloid PET. (2) Cox proportional hazard regression revealed that only modified RCTU sum score was a significant covariate. (3) It can also provide symmetry or asymmetry deposit of amyloid. Patients with symmetric deposits of amyloid showed a significantly higher risk for AD conversion.

Due to the requirement for specialized software and expertise, quantitative evaluation of amyloid PET/CT using SUVR may be challenging in routine clinical practice. We introduced the RCTU ‘sum’ scoring, aggregating RCTU scores in the hope that it would serve as a semi-quantitative value for evaluating and predicting AD conversion. Both conventional and modified RCTU sum scores correlated well with amyloid SUVR whole brain (Figure 3a,b). Additionally, we conducted a ROC analysis; however, no statistical difference was observed in the ROC AUC between conventional and modified RCTU sums (refer to Section 3.8 of the results). However, in the Cox proportional hazard regression analysis, only the modified RCTU score emerged as a significant covariate. In addition, not only the ‘sum’ of the scores but also the individual scores of the modified RCTU for each lobe were well correlated with the amyloid SUVR value. (Figure 3), suggesting that the modified RCTU score can be used more usefully as a semi-quantitative value than the conventional RCTU score.

A significant advantage of the modified RCTU score is its ability to provide information on left/right asymmetry or symmetry in amyloid deposits. The conventional RCTU does not differentiate between the left and right lobes of the brain. For instance, the conventional RCTU does not account for differences in cases where amyloid deposition occurs in the bilateral frontal lobes versus when it is present in just one frontal lobe. The rationale for modifying the conventional RCTU score was based on the evidence that amyloid deposition in the brain is not always symmetric between the hemispheres [23]. By incorporating the right and left hemispheres separately, the modified RCTU score provides a more comprehensive and sensitive measure of amyloid burden in aMCI patients. This nuanced approach of assessing each lobe individually may provide more useful insights into the progression from aMCI to AD. We could determine the symmetry of amyloid deposits using modified RCTU scores. As shown in our study, symmetry in amyloid deposits was a significant risk factor for AD conversion, highlighting the usefulness of knowing a patient’s bilateral amyloid deposit status—a feature not available with the conventional RCTU.

To our knowledge, this is the first study that used modified RCTU variables in the analysis of amyloid PET for AD prediction. Cox proportional hazard regression analysis showed that not the sum of the conventional RCTU score but the modified RCTU sum score was significant in AD conversion. The hazard ratio (Exp(B)) was calculated to be 1.1318, indicating a 13.2% increase in the risk of AD conversion for each unit increase in the modified RCTU sum.

The present study adds to the growing body of evidence supporting the utility of amyloid PET imaging in predicting AD conversion in aMCI patients. Our findings are consistent with previous studies, including a recent 4-year follow-up study which demonstrated that 37.5% with positive amyloid PET patients converted to AD over 4 year period compared to 5.3% with negative amyloid PET patients [24].

Another limitation of this study is the relatively small sample size, which may limit the generalizability of the findings. In conducting the survival analysis, we utilized the duration until the diagnosis of AD conversion. However, there is a major drawback in that the duration can be assessed differently depending on the starting point of observations and also on interval of observations. Future studies with larger and more diverse samples are needed to confirm the utility of modified RCTU scores in AD prediction and monitoring. Future studies should include such outcomes to validate the clinical significance of modified RCTU scores.

## 5. Conclusions

In conclusion, our study suggests that the modified RCTU score, in its role as a semi-quantitative, visual scoring tool, shows promising efficacy in evaluating amyloid deposits and, therefore, in predicting AD conversion. To further validate these findings and optimize the scoring method, future studies are warranted with larger sample sizes and extended follow-up periods.

## Figures and Tables

**Table 1:** Patient characteristics.

*n*	45
Age (mean ± SD, y)	76.24 ± 7.12
Sex (F:M, n)	19:26
Follow up interval (mean ± SD, month)	23.62 ± 9.11
Education (mean ± SD, (range), y)	9.28 ± 4.74
MCI domain (single/multi domain, n)	12:33
Right handedness (n)	44
apoE4 ^a^ (carrier/non-carrier, n)	16:28
apoE4 heterogeneity (non-carrier: homozygotes: heterozygotes, n) ^a^	28:3:13
K-MMSE (mean ± SD)	24.87 ± 2.81
Amyloid scan positivity (positive: negative), n	17:28

a: one subject refused to test.

**Table 2:** Comparison between AD converter group and non-converter group.

	AD Converter Group (*n* = 12)	Non-Converter Group (*n* = 33)	*p* Value
N (aMCI)	12	33	
Age (mean ± SD, (range), y)	74.5 ± 9.59 (63–89)	76.88 ± 6.03 (67–89)	0.435
Sex (F:M, n)	4:8	15:18	0.4716 ^a^
Follow up interval (mean ± SD, (range), month)	21.07 ± 8.51 (11.7–34.1)	24.55 ± 9.27 (11.63–41.3)	0.262
Time to AD Conversion Diagnosis from First Visit (mean ± SD, (range), month)	17.8 ± 6.85 (12.1–30.1)	NA	NA
Education (mean ± SD, (range), y)	10.01 ± 5.38 (0–17)	8.99 ± 4.54 (0.5–18)	0.498
Right handedness (n)	12	32	1 ^c^
apoE4 (carrier:non-carrier, n) ^b^	5:6	11:22	0.4743 ^a^
apoE4 heterogeneity (non-carrier: homozygotes: heterozygotes, n) ^b^	6:1:4	22:2:9	0.3445 ^a^
Positive scan: negative scan (n)	7:5	10:23	0.086 ^a^
amyloid SUVR whole brain ^d^, median (range)	1.14 (1.02–1.43)	1.22 (0.97–1.46)	0.190
Conventional RCTU variables, median (range)			
conventional RCTU_Sum ^d^	9.5 (4–12)	4 (4–12)	0.019 *
conventional RCTU_Frontal ^d^	3 (1–3)	1 (1–3)	0.017 *
conventional RCTU_Parietal ^d^	3 (1–3)	1 (1–3)	0.032
conventional RCTU_Temporal ^d^	2.5 (1–3)	1 (1–3)	0.032
conventional RCTU_PC2 ^d^	1 (1–3)	1 (1–3)	0.004 *
BAPL scoring, median (range)	3 (1~3)	1 (1~3)	0.047 *
Modified RCTU variables, median (range)			
Modified RCTU Sum (range)	18 (7–21)	7 (7–21)	0.018 *
Modified RCTU left sum ^d^	8.0 (3–9)	3.0 (3–9)	0.032 *
Modified RCTU right sum ^d^	8.5 (3–9)	3.0 (3–9)	0.032 *
Modified RCTU_Frontal_Left	3 (1–3)	1 (1–3)	0.009 **
Modified RCTU_Frontal_Right	3 (1–3)	1 (1–3)	0.009 **
Modified RCTU_Parietal_Left ^d^	3 (1–3)	1 (1–3)	0.017 *
Modified RCTU_Parietal_Right ^d^	3 (1–3)	1 (1–3)	0.017 *
Modified RCTU_Temporal_Left ^d^	2 (1–3)	1 (1–3)	0.009 *
Modified RCTU_Temporal_Right	2.5 (1–3)	1 (1–3)	0.017 *
Modified RCTU_PC2	1 (1–3)	1 (1–3)	0.005 *

*: <0.05, **: <0.005; ^a^: chi-square test; ^b^: one subject refused to test; ^c^: Fisher’s exact test; ^d^: M-W test; Abbreviation: aMCI, amnestic minor cognitive impairment; AD, Alzheimer’s disease; NA, not applicable; BAPL, brain amyloid plaque load; RCTU, regional cortical tracer uptake; PC2, posterior cingulate cortex/precuneus.

## Data Availability

The datasets generated during and/or analyzed during the current study are not publicly available due to internal research policy decisions.
